# Tribological and antioxidation properties study of two N-containing borate ester derivatives as additive in rapeseed oil

**DOI:** 10.1371/journal.pone.0207267

**Published:** 2018-12-10

**Authors:** Zhongyi He, Liping Xiong, Feng Xie, Mingxue Shen, Sheng Han, Jianqiang Hu, Wenyuan Xu

**Affiliations:** 1 School of Materials Science and Engineering, East China Jiaotong University, Nanchang, China; 2 Department of Aviation Oil and Material, Air Force Logistics College, Xuzhou, China; 3 Shanghai Applied Technology University, Shanghai, China; University of Waterloo, CANADA

## Abstract

Two kinds of phenol- and N- containing borate ester, BTEB and BMEB have good hydrolysis stability due to the B-N coordination bond. The P_*B*_ value improved by 60.7% and 67.6% respectively at 0.5wt% BTEB, BMEB in rapeseed oil. Their antiwear effect increases with the increase of adding content, and BMEB is better than BTEB. The friction-reducing effect of BTEB is better than BMEB. All additives formed a protective film which containing BOx, FeOx and other organic nitrogen compounds. The better capacities of BMEB may due to the complex boundary lubricating film which contain ferrous sulfate, ferrous sulfide. All additives possessed good antioxidation effect, and it increased the oxidation activation energy than rapeseed oil by 51.15% and 78.82% respectively at 0.25wt%.

## Introduction

The traditional phosphorus-containing lubricant is restricted to use, so novel additive with efficient, phosphorus-free, ashless, antiwear and antioxidation ability [1] is the research focus. Borate ester [2] is considered to be such a novel additive, for not only its special extreme pressure, oxidation stability and good anticorrosion, sealing performance, but also its toxic-free and fetid odor, and some of it’s derivatives have been used in gear oil and two stroke oil [3]. Its tribology property, like extreme pressure and antiwear ability, will come to be poor when use alone, for easy hydrolysis [4], because of the empty 2*p* orbit of B atom easy attacked by the lone pair electrons, such as oxygen atom in water molecule, and decreased it’s stability[5], so it is difficult to store for long time.

Now, research mainly focused on introduction of electron-rich groups (such as nitrogen atom) into borate ester, to stabilize the electron-deficient of boron atom[6], and that can reduce the possibility of attack by some nucleophiles. More importantly, it can enhance antiwear performance [7] for the synergetic effect between boron and nitrogen element. It improved the hydrolytic stability of borate esters by adding some amine compounds to form the extra-molecular-coordination bond, which was revealed by Small [8] and Papay [9]. Zheng [10] and Yao [11] introduced imido and aminoethyl groups into borate ester with a stable six- and five-member ring structure, which improved the hydrolytic stability of as-synthesis additives for making use of the intra-molecular- coordination between nitrogen and boron atom. So these N-containing borate esters are showing great potential as a new generation of green lubricant additive.

Guangbin Yang[12] has synthesized a kind of N-containing borate ester (DEBE) with double five-member-ring structure as RSO additive by using boric acid, diethanolamine and alkylphenol polyoxyethylene ether as starting materials. Test results show that the as-prepared borate ester possessed good antiwear properties and hydrolysis stability, and they can be used as a promising sulfur-and phosphorus-free environmentally acceptable lubricating oil additive. In our previous research[13], an N-containing borate ester (BMB), which containing a five- member ring and a seven-member ring structure, possesses good tribology and hydrolysis stability, for the forming a coordination bond between the lone electron of nitrogen atom with boron atom. When N-containing heterocycle group [4,7] (such as mercaptobenzothiazole benzotriazole, etc.) was introduced into organic borate ester molecule, the electron-rich group can effectively improve it’s hydrolysis stability.

Antioxidant can greatly improve oxidation stability[11] of lubricating oil, prevent oil deterioration and prolong the oil change period[14]. An additive which containing shielding phenol and dialkyl amine, has expressed good performance in capturing free radicals and decomposition of hydrogen peroxide, in addition, it also improve the oxidation stability of lubricating oil.

Sulfur and/or nitrogen-containing heterocyclic compounds[15], such as benzotriazole, thiadiazole and mercaptobenzothiazole derivatives, can inhibit the catalytic effect of metal in the oxidation process[13], to improve its antioxidation effect. At the same time, they have the function of inhibiting metal corrosion, and they usually have been used in industrial oil. A benzotriazole-type metal deactivator [16] significantly increased the test time of rotary oxygen bomb with 0.02 wt% to 0.5wt% content as mixed with 2,6-di-tert-butyl-p-cresol (BHT), and expressed good intermolecular synergistic effect on antioxidation.

In the present research [4], we select sterically hindered phenol with long carbon chain, boric acid and 2-(benzotriazol-1-yl) ethan-1-ol (code as BTE), 2-(benzimidazole-2-ylthio) ethan-1-ol (code as BME) as staring materials to synthesize two P-free borate ester BTEB and BMEB, with the aim to improve the tribological performance and hydrolytic stability of borate ester. This article reports the details about the preparation and evaluation of the tribological properties in relation to worn surface analysis by scanning electron microscope (SEM) and x-ray photoelectron spectroscopy (XPS). Their antioxidant effects and mechanism were analysis by using the pressure differential scanning calorimetry (PDSC) analyzer.

## Methodology

### Materials

The 4-dodecylphenol is industrial grade, BTE and BME are prepared in the laboratory. Light green liquid, 2-((dibuthylamino)methyl)-4dodecyl-5-(hydroxymethyl)phenol (coded as DDP) were prepared as the article [17], others are chemical grade reagents shopped from Shanghai Chemical Company. The natural rapeseed oil was used as base oil, produced by Fuzhou Co. Ltd of China. Its physical and chemical properties are as follows: total base numbers (TBN) is 0.78 mg KOH/g, viscosity index is 211, υ40°C is 37.93 and basic nitrogen content is 0.186 mg/g. All reagents were used directly without further treatment.

### Additive synthesis

**Fig 1:**
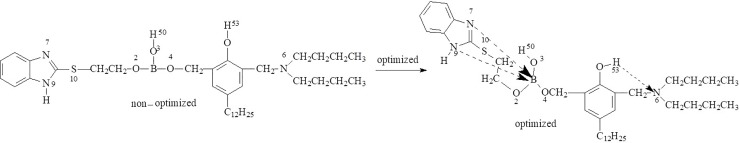
Synthesis route of compounds.

The synthesis route of additives is shown in Fig 1. The specific process is as follows: 200 mL toluene, 0.1mol BTE or BME, 0.1mol DDP and 0.1mol boric acid were added in a 500 mL flask with water-separator and stirring at room temperature. The reactant was quickly heated up to 110°C and refluxed for 3h in a nitrogen atmosphere with acid resin as catalyst, followed by cooling to room temperature when the produced water reached the theoretical amount. Finally through filtration, drying and vacuum distillation, 59.4g yellow transparent liquid, 2-(benzotriazol-1-yl) ethyl (3-((dibutylamino) methyl) -5-dodecyl-2-hydroxybenzyl) hydrogen borate (coded as BTEB) was obtained with a yield of 88.6%. Or 45.4g yellow transparent liquid, 2-(benzoimidazol-2-ylthio)ethyl (3- ((dibutylamino)methyl) -5-dodecyl-2- hydroxybenzyl) hydrogen borate (coded as BMEB) was obtained with a yields of 73.6%.

### Structure characterization

The chemical structure of BTEB and BMEB were analyzed with Infrared (IR) spectra by using a Spectrum One type Fourier transform infrared (FT-IR) spectrometer (Perkin Elmer Instruments Co. Ltd, USA). The C, H, N, S elements content were analyzed by vario EL III type element analysis instrument, and the content of B element was determined by a Varian 720-ES ICP. Thermochemical stability was detected by Pyris 1 TGA 4000 type (Perkin Elmer Instruments Co. Ltd, USA) at a nitrogen environment with a flow of 40 ml / min, the temperature range from 50°C to 750°C, with a heating rate of 10°C/ min.

### Hydrolysis stability

Hydrolysis stability was investigated by the half-life method [4]. 50 ml water and 5 ml glycerin were added in a glass, then mixed with a lot of 0.1 mol/l NaOH solution, and 3 to 4 drops phenolphthalein solution as indicator. This mixture was added into a beaker with 0.4~1.2 mmol as-prepared additive (twice amount of NaOH). The solution was stirred continuously until the color changed from red to colorless. The changing time was considered the hydrolysis stability time.

### Computing method

All the calculations were completed at the Eastchina Jiaotong University computing workstation. The geometrical configuration of borate ester[13] was optimized with a program of Gaussian 09, in B3LYP/6-31G* level. The charge quantity and atom distance of additives were calculated to obtain the reasonable space structure.

### Tribological property test conditions

The tribological property of BTEB and BMEB were carried out by a MMW-1 type four-ball machine (made in Ji’nan Shunmao Factory, China) at room temperature, 1450 rpm for 30 min, according to GB3142-82 (Chinese national standard, similar to ASTM D-2783). The load-carrying cability (P_B_ value) was measured at 1450 rpm for 10 s. All test balls (Φ = 12.7 mm) are made of GCr15 bearing steel (C, 0.95–1.05%; Si, 0.15–0.35%; Mn, 0.20–0.40%; P,<0.027%; S, < 0.020%; Cr,1.30–1.65%; Ni, < 0.30%; Cu, < 0.25%) with an HRc59–61. Before each test, the specimens were cleaned in petroleum ether, and then dried. A microscope was used to determine the wear scar diameters (WSD) of the three lower balls with an accuracy of 0.01 mm, and the friction coefficient was recorded automatically.

### Worn surface analysis

The tested steel balls were ultrasonicly cleaned 15 minutes in petroleum ether (60–90°C). Vega3 type SEM (made in Tescan Company) and Zeiss Sigma EDS analysis were used to investigate the surface morphology and elemental distributions on the worn surface respectively. The chemical states of typical elements on the worn surfaces were analyzed by using a PHI-5702 type XPS analyzer. The binding energy was calibrated by C1s = 284.6 eV.

### Antioxidation property

The initial oxidation temperature (IOT) and oxidation induction time (OIT) of oil samples were investigated by DSC8000 type (Perkin Elmer Instruments Co.Ltd company) PDSC tester with temperature programming method and constant temperature method. And the measuring conditions: temperature range from 50°C to 350°C, heating rate 40°C / min at the oxygen ambience.

## Results and discussion

### Structure analysis

**Fig 2:**
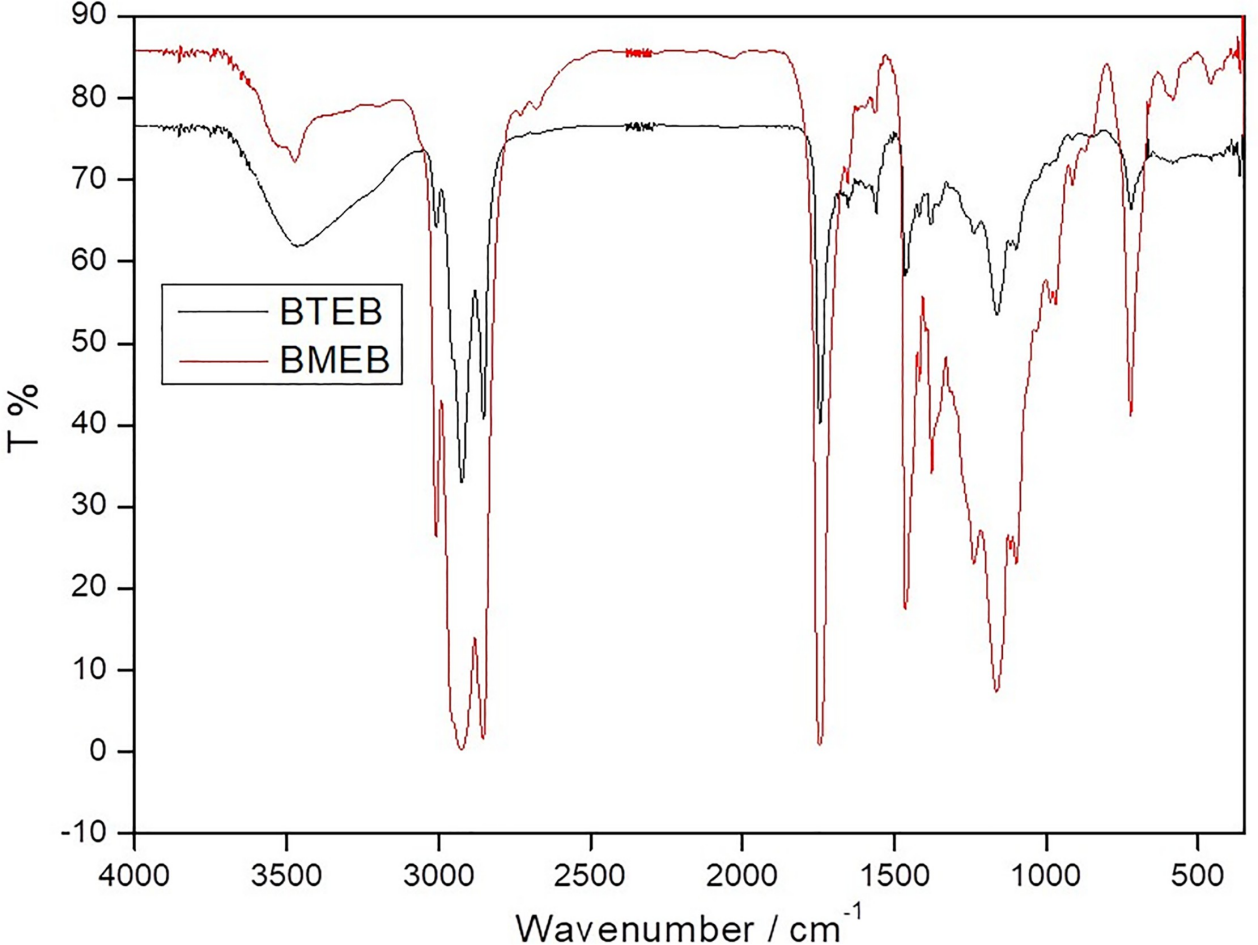
IR spectra of additives.

The infrared analysis results of BTEB and BMEB are shown in Fig 2. It can be seen that the peak at 3301 cm^-1^ (BTEB), 3201 cm^-1^ (BMEB) are the stretching vibration absorption peak of phenolic hydroxyl group, the peak at 2959 cm^-1^ (BTEB), 2958 cm^-1^ (BMEB) are the C-H bond stretching vibration peak, and the B-O (belongs to borate ester) stretching vibration absorption peak is at 1378 cm^-1^ (BTEB), 1378 cm^-1^ (BMEB), and the -C-N- absorption peak appears at 1020 cm^-1^. All characteristic peaks appeared on the infrared spectrum.

**Fig 3:**
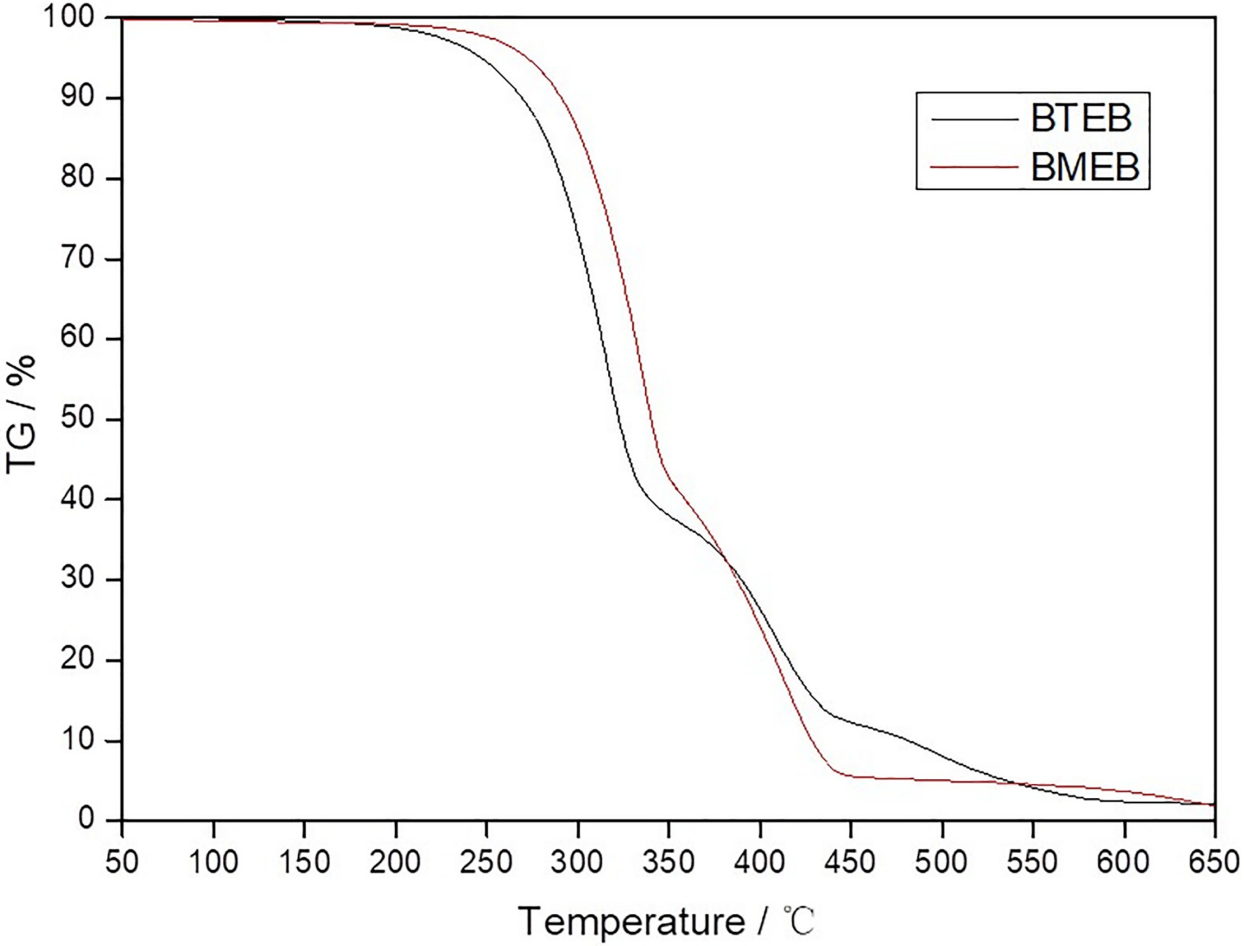
The TGA curve of additives.

The thermal stability analysis results are shown in Fig 3. The BTEB and BMEB have excellent thermal stability, and the initial decomposition temperature reach 207°C and 220°C respectively, which meet the thermal stability requirement of lubricating oil additive.

Their elemental analysis results are listed in Table 1, and the data in parentheses are theoretical values. The measured values are in good agreement with the theoretical values, within the experimental error.

**Table 1:** Element content analysis of the synthesized compounds.

Compd	C(%)	H(%)	N(%)	S(%)	B(%)
BTEB	72.49(72.48)	9.68(9.40)	8.39(8.58)	0(0.00)	2.12(2.02)
BMEB	66.89(66.27)	8.62(8.81)	4.27(4.18)	9.40(9.55)	1.58(1.54)

It can be found that the synthesized products are the target borate esters according to the infrared spectrum, element content analysis results.

### Hydrolysis stability

The turbidity time of 1.0wt% trimethyl borate and 1.0wt% tributhyl borate (TBB) in petroleum wax oil were only 5 min and 10 min respectively. The turbidity of petroleum wax oil with 1.0 wt% BTEB / BMEB did not change after stored six months at ambient temperature. These indicated that the as-prepared BTEB and BMEB additives possessed excellent storage stability, due to their special structure.

In order to short the turbidity time, 0.2 g water was added to these samples and with a constant temperature (70°C), and the turbidity time was marked as the hydrolysis time. The samples components and hydrolysis time are shown in Table 2. The hydrolysis time of BTEB, BMEB were 83678 s and 103257s respectively, which was more 1,300 times and 1600 times than that of TBB respectively, and they are also bigger than the mixture of BTE (BME) and TBB at any mass ratio. Thus, to make up for the B electron deficiency, the N-containing ethanol within borate ester by inner coordination was better than that of the outer coordination with TBB.

**Table 2:** Samples components and hydrolysis time.

Samples	Paroline	H_2_O	TBB	BTE or BME	BTEB or BMEB	Time/s		Deviation time(s)
						BTEB	BMEB	
Black	24.80g	0.20g	0.25g	0.00g	0.00g	63	63	±1
A	24.80g	0.20g	0.25g	1.00g	0.00g	386	231	±1
B	24.80g	0.20g	0.25g	0.75g	0.25g	10231	836	±6
C	24.80g	0.20g	0.25g	0.50g	0.50g	66353	23056	±6
D	24.80g	0.20g	0.25g	0.25g	0.75g	80252	57889	±6
E	24.80g	0.20g	0.25g	0.00g	1.00g	103257	83678	±6

### Hydrolysis stability mechanism

#### The atoms charge analysis

**Fig 4:**
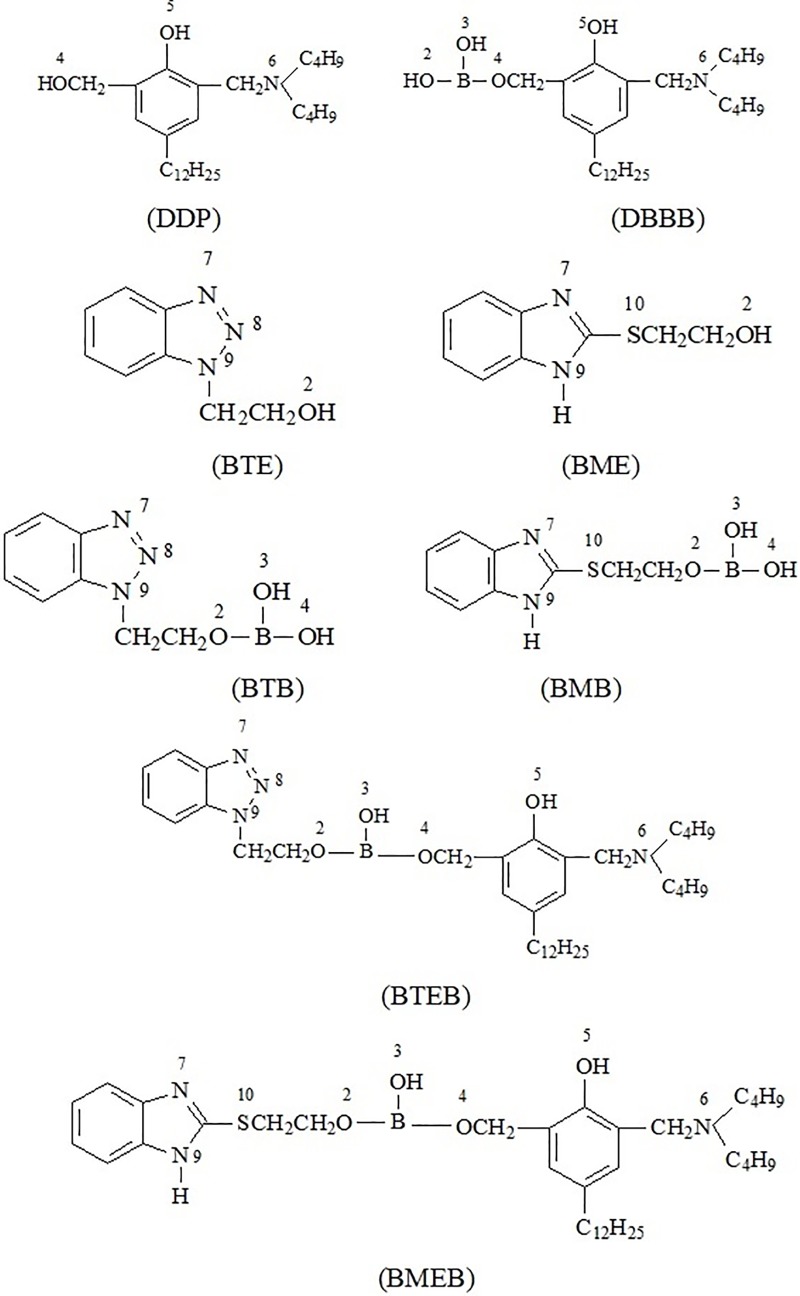
Structure, atom label and code of compounds.

The structure, code and atom label of these compounds are shown in Fig 4. And the Mulliken charges of nitrogen, boron, oxygen and sulfur atom in the as-prepared borate esters and their derivatives are shown in Table 3 and Table 4.

**Table 3:** The Mulliken charge of different atoms in BTEB and it’s derivatives.

Elements	H_3_BO_3_	BTE	DDP	DBBB	BTB	BTEB
B	0.688664			0.733956	0.735443	0.783559
O(2)	-0.558724	-0.551515		-0.571206	-0.532812	-0.539018
O(3)	-0.530062			-0.566863	-0.537422	-0.583809
O(4)	-0.564066		-0.523892	-0.509344	-0.547594	-0.535678
O(5)			-0.548896	-0.598157		-0.616496
N(6)			-0.500937	-0.438860		-0.405273
N(7)		-0.361817			-0.362285	-0.365035
N(8)		-0.048376			-0.05693	-0.050120
N(9)		-0.410935			-0.422541	-0.409956

**Table 4:** The Mulliken charge of different atoms in BMEB and it’s derivatives.

Elements	H_3_BO_3_	BME	DDP	DBBB	BMB	BMEB
B	0.688664			0.733956	0.733311	0.785775
O(2)	-0.558724	-0.531626		-0.571206	-0.524167	-0.519342
O(3)	-0.530062			-0.566863	-0.537238	-0.562892
O(4)	-0.564066		-0.523892	-0.509344	-0.548185	-0.492286
O(5)			-0.548896	-0.598157		-0.594318
N(6)			-0.500937	-0.438860		-0.513228
N(7)		-0.506885			-0.511705	-0.606201
N(9)		-0.532449			-0.606685	-0.511828
S(10)		0.193100			0.146983	0.171506

Comparing the charge of nitrogen atom between BTEB (BMEB), BTE (BME) and DDP, DBBB, it was found that the charge of nitrogen atom (labeled 6) in different aniline groups have changed. The charge of nitrogen atom in DDP is -0.500937, but they are -0.405273 and -0.513228 in BTEB and BMEB respectively.

The positive charge of boron atom in BTEB (0.783559) is less than that of tributhyl borate (0.802498) due to the electron withdrawing effect of DDP. The positive charge of B atom in BMEB (0.785775) is greater than BMB (0.733311), and that in DBBB is similar to that in BMB (BTB), they are all near 0.733. The positive charge of the B atom in BTEB (BMEB) is around 0.78, which is larger than that of DBBB and BMB (BTB), indicating that the B atom is electron-deficient in the synthetic additive, but it’s not very different, and the electron-deficient lead to the decrease of hydrolysis stability of the additives. When boron atoms are connected with N-containing heterocycles, the electron-deficient of boron atom are increased. But the increase is not much, only from 0.73 to 0.78, so there is little effect on the electron-deficient of B atom.

The Mulliken charge of oxygen atom is -0.435597 in glycol, and it is -0.619572 in water, so the nucleophilic of water is bigger than glycol. By comparing that in BTEB and BMEB, the lowest Mulliken charges of nitrogen atoms in BTEB and BMEB are -0.409956 and -0.606201 respectively. And the nucleophilic of borate esters is lower than glycol and water. The nucleophilic activity of N (labeled 7) in BMEB is near the nucleophilic of oxygen in water, and that of N (labeled 9) in BTEB is near the nucleophilic of oxygen in glycol, so the two atoms are more likely to be closer to the boron atom.

#### Separation distance between B atom and different N atom

Table 5 shows the bond length of B-O bond in different compounds after optimized, we know that the bond length in different compounds are all around 1.640Å before optimized, which is almost equal to the B-O bond length in boric acid. There were little different between before and after optimization, and it means that the introduction of other functional groups into boric acid molecule has little effect on the bond length of B-O bond.

**Table 5:** The bond length of B-O bond after optimized structure.

	B-O(2)	B-O(3)	B-O(4)
Boric acid / Å	1.641	1.641	1.641
TBB/ Å	1.635	1.653	1.641
BTEB/ Å	1.646	1.646	1.643
BMEB/ Å	1.643	1.653	1.647

**Fig 5:**
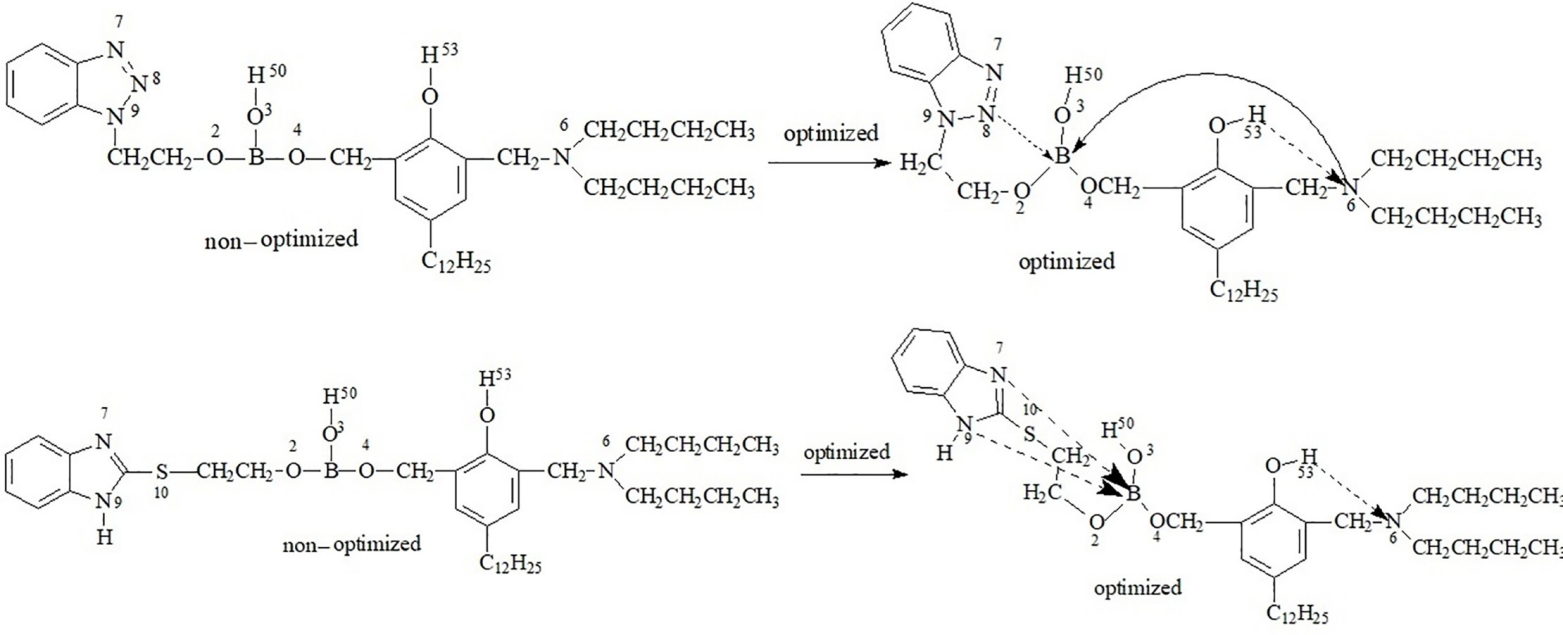
Stable structure of BTEB and BMEB.

The space distance between B and all N atoms were shown in Table 6. For BTEB, the distances between nitrogen atoms and boron atom all become shorter after optimization. In particular, the distance between boron atom and N (8), N (6) atoms are changed from 5.770Å to 5.137Å and from 6.479Å to 4.629Å respectively, and that means the boron atom formed a six-membered ring and a eight-membered ring with the nitrogen atom on the benzotriazole ring and the methylamino group respectively through coordination bond. For BMEB, the distances between N(9), N(7) atoms and boron atom also become shorter after optimization, it is from 5.087Å and 6.556Å to 4.329Å and 6.154 Å respectively, and it also means that two seven-membered rings were formed between boron atom and N(9) atom with a coordination bonds. That supported their stable structure in Fig 5. It means that there are two rings that make the boron and nitrogen atoms form a coordination bond after the formation of the molecule, and these make borate ester more stable and not easy to hydrolyze.

**Table 6:** Separation distance between B atom and different N atoms.

	B-N(7)	B-N(8)	B-N(9)	B-N(6)
BTEB(non-optimized) / Å	4.971	5.770	6.732	6.479
BTEB(optimized) / Å	4.874	5.137	6.476	4.629
BMEB(non-optimized) / Å	6.556		5.087	6.000
BMEB(optimized) / Å	6.154		4.329	6.018

The space distance between hydrogen atom in hydroxyl group and all nitrogen atoms were shown in Table 7. In DDP and DBBB molecules, a six-membered ring is also formed between H(53) and N (6) in the form of intramolecular hydrogen bonds. The distance between H(53) and N(6) in DDP (DBBB) molecules are more than 3.0 Å, but it becomes smaller when connected with borate, the distance between them is closer. From Table 8, a six-membered ring has formed between H(53) and N(6) with an intramolecular hydrogen bond, because the distance becomes from 3.768Å to 2.158Å. These ring structures increase the steric hindrance of BTEB, thus improving its hydrolysis stability. For BMEB, a six-membered ring is also formed between H(53) and N(6) through a intramolecular hydrogen bond. These rings build a structure that puts boron atom in the center of borate ester. The oxygen and nitrogen atoms in water-glycol fluid are not easy to attack the electron-deficient boron atom, thus improving the hydrolysis stability of borate ester. When the DDP functional group connected with the N-containing heterocyclic group to form borate, they do not destroy their stable structure, even N(6) atom and boron atom form a coordination bond. And a six-membered ring with a intramolecular hydrogen bond is formed to make the molecule more stable.

**Table 7:** Separation distance between H atom and different N atoms.

	H(53)-N(7)	H(53)-N(6)	H50-N(7)	H50-N(6)
BTEB(non-optimized)/ Å	4.466	3.769	7.152	8.078
BTEB(optimized)/ Å	5.875	2.158	8.100	8.052
BMEB(non-optimized)/ Å	7.445	3.807	7.321	7.760
BMEB(optimized)/ Å	8.755	3.173	6.380	7.359
DDP (non-optimized)/ Å		3.945		
DDP (optimized)/ Å		3.173		
DBBB (non-optimized)/ Å		3.868		
DBBB (optimized)/ Å		3.173		

**Table 8:** The P_B_ values of oil samples at different concentrations.

Samples		0.0%	0.1%	0.3%	0.5%	0.7%	1.0%
P_*B*_ / N	BTEB	696	932	981	1020	1020	1058
	BMEB	696	932	1069	1138	1138	1138

For BTEB molecule, the distance between H(50) and N(7) changed from 7.152Å to 8.100Å, they are moved further apart. For BMEB molecule, the distance between them changed from 7.321Å to 6.380Å, and the distance became smaller. It shows that N(7) has a certain attraction to H(50), because the electronegativity of nitrogen element is very large.

From the above analysis, the Gaussian calculation results showed that the hydrolysis stability of BTEB (BMEB) may be attributed to the lone electron pair of nitrogen atoms in heterocyclic ring, which can form a coordination effect with boron atom to compensate for the shortage of electrons of the boron atom, as seen from the charge of the B and N atoms, as well as the distance between the N atoms and B atom.

The root cause of easy hydrolysis of borate ester is the empty 2*p* orbital in boron atom by *sp2* hybridization [18]. The empty 2*p* orbital is easily attacked by nucleophile groups, which has lone electron pair. The oxygen atom in water and glycol all contain lone electron pair, which can attack the B atom to make borate ester hydrolyze into boron acid. According to many extant researches, the hydrolysis process of borate ester is accomplished in three steps: first, the borate ester is attacked by water, then the unstable tetrahedral complex is generated, and finally, alcohol is desquamated.

When the structure of borate ester has changed, the negative charge of boron atom is also changed, but the hydrolysis stability is increased according the results in Table 2, it is mainly caused by its spatial structure change, resulting in huge steric hindrance. However, the spatial structure changes, and the larger functional groups of DDP have larger steric hindrance. The attack of water on boron atom is reduced and its hydrolytic stability is improved.

**Fig 6:**
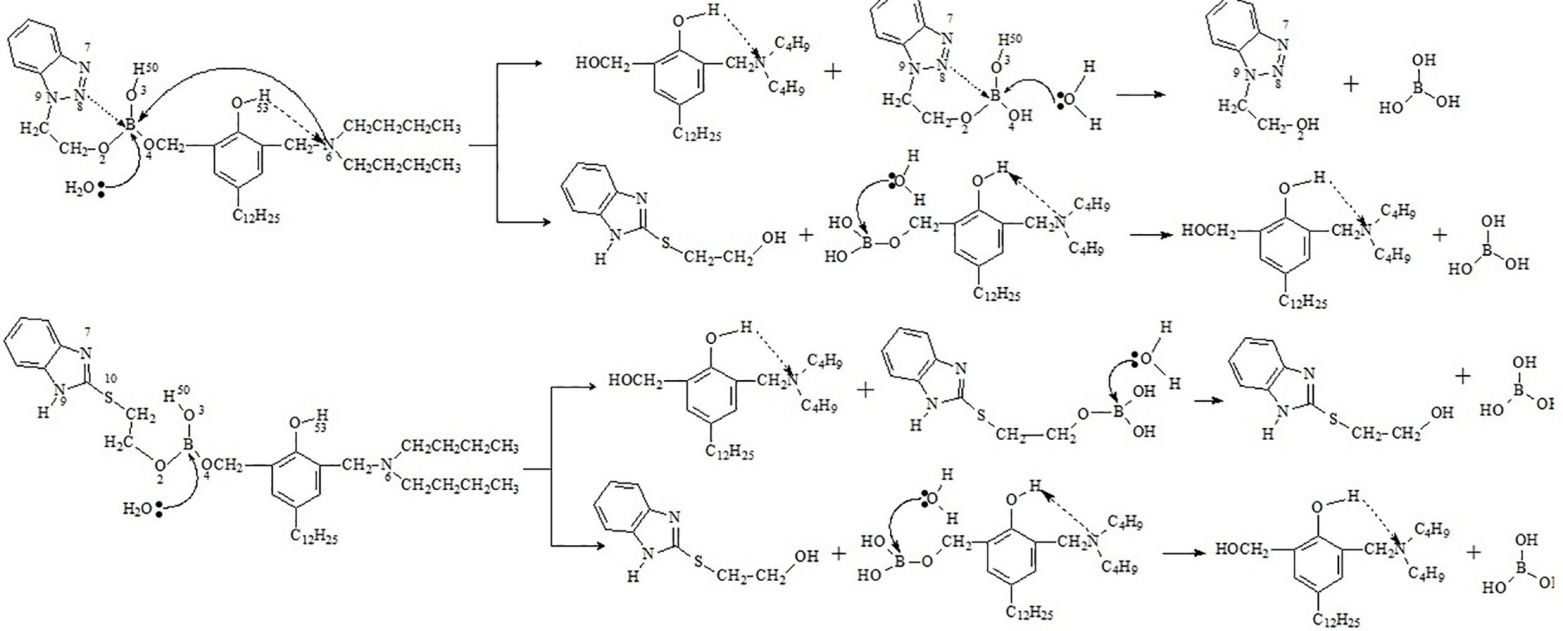
The hydrolysis process.

Due to the positive charge of B atom in BTB (BMB) were less than BTEB (BMEB), so the hydrolysis stability of hydrolyzed products in the first step is higher, but the positive charge of B atom of the two hydrolysates is similar, indicating that all the two hydrolysis processes exist. According to the calculation and analysis results, the hydrolysis process may be shown as the below Fig 6.

### Load-carrying properties

The extreme pressure property (P_*B*_ value) of different oil samples were shown in Table 8. The P_*B*_ values taken on an upward trend along with increase of additives content. When BTEB and BMEB were 0.5wt%, it enhanced the P_*B*_ value of RSO more than 46.6% and 63.5% respectively. The P_*B*_ value increased slowly when additive content was more than 0.5wt%, and it cann’t make P_*B*_ value a rapid increase as further increase of additive content. At the same content, the P_*B*_ value of BMEB is greater than BTEB, because of actively sulfur in BMEB. The BMEB molecule adsorbed on the friction pairs surface, and sulfur reacted with the metal surface to form a triboreaction film in a relatively depth of matrix, and this film more improved the extreme pressure property of RSO.

### Antiwear properties

**Fig 7:**
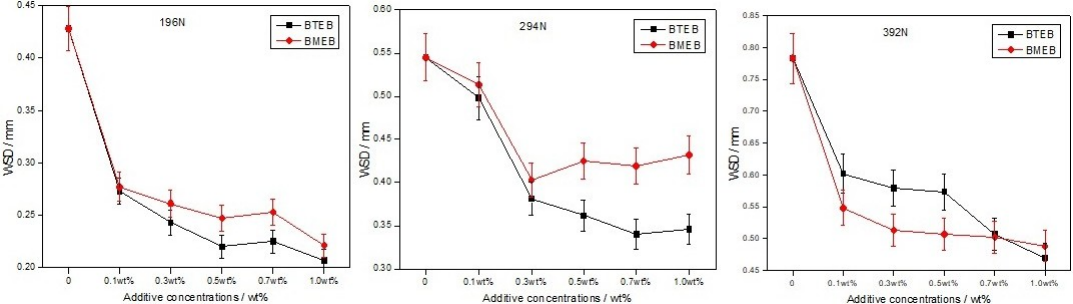
WSD changes with additive content. (left) 196N;(middle) 294N; (right) 392N.

The wear scar diameters (WSD) of BTEB and BMEB at different loads are shown in Fig 7. The WSD were decreased along with the increase of additive content from 0.10wt% to 1.0wt% in all applied loads, and the as-prepared borate esters improved the antiwear property of RSO at all concentrations.

In low load (196N), for BMEB, the smallest WSD was 0.221 mm at 1.0 wt% adding content, much smaller than that of pure oil (0.428 mm). The WSD value decreased rapidly when 0.10 wt% BTEB was added in RSO, and the minimum WSD was 0.207 mm at 1.0 wt% content, also much smaller than that of RSO. Under 196N and 294N, the antiwear effect of BTEB is better than that of BMEB, which is related to the strength of its physical adsorption film [19]. The large π bond [20] of benzotriazole makes BTEB more easier to adsorb on the metal surface, and form a dense adsorption film, which has good antiwear effect.

While in high load (392N), the antiwear effect of BMEB was more superior at higher contents. The antiwear ability of BMEB was better than BTEB at the same condition, the different antiwear ability between them may due to their different molecular structure, for BMEB contains actively sulfur element, and these indicates that sulfur is a main index of good antiwear property for this kind of additive in high applied load.

### Friction-reducing properties

**Fig 8:**
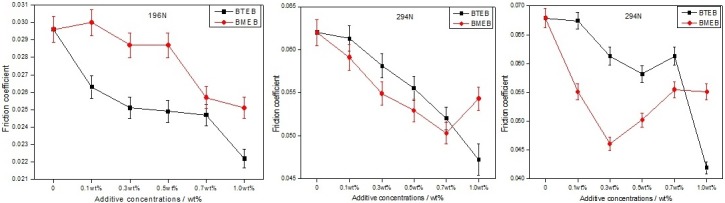
Friction coefficient changes with content, (left) 196N;(middle) 294N; (right) 392N.

Fig 8 represent the relationship between friction coefficient and additives content under different loads. It can significantly reduce the friction coefficient of oil samples by adding additives in pure oil. The friction-reducing effect of BTEB is better than BMEB under 196N, and less than BMEB under 392N. Under low load, the BTEB formed a layer more effect physical adsorption film on the contact surface, because the big π bond strength of BTEB is stronger than BMEB, and the film has friction-reducing property. Under high load, chemical reaction film formed on the worn surface because of the friction heat, the ability to form a chemical reaction film of BMEB is stronger than BTEB because of the actively sulfur, so lead to good friction-reducing performance. In 294N load, the friction coefficient of BTEB is smaller than BMEB when the adding concentration is less than 0.7wt%, while the adding concentration is more than 0.7wt%, the friction-reducing performance of BMEB is better than BTEB, but they are little different. This means that under moderate load, the friction-reducing properties of the two additives are similar, which are related to their similar chemical structure, and the friction-reducing performance of the mixed boundary film is also similar.

### SEM analysis

**Fig 9:**
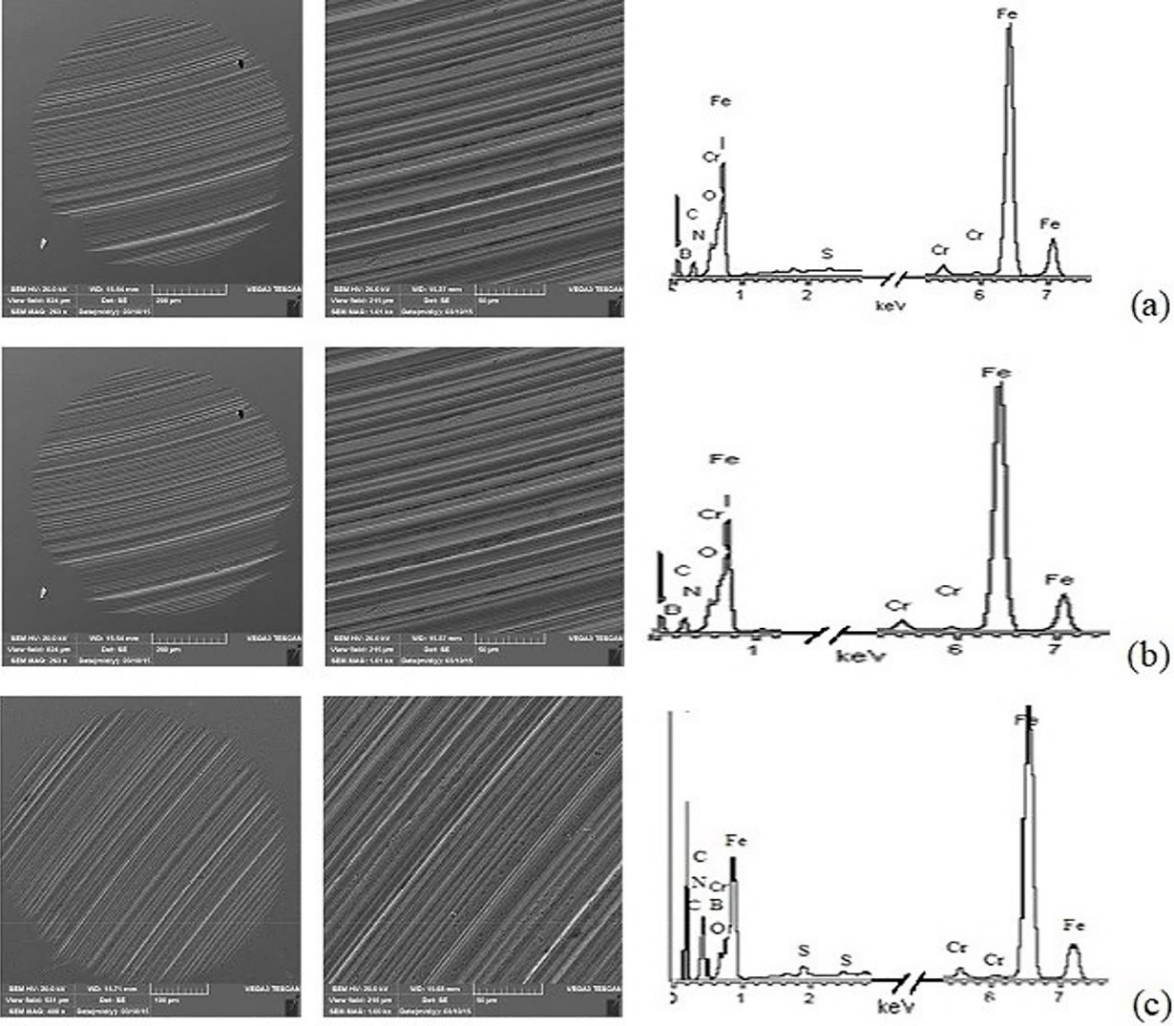
The SEM photos and EDS analysis, (a) RSO; (b) 0.3wt% BTEB; (c) 0.3wt%BMEB.

Fig 9 showed the SEM images and EDS analysis of the worn surface lubricated with pure oil, 0.3wt% BTEB and 0.3wt% BMEB respectively at 392 N, 1450 rpm for 30 min, and the element content results of worn surface are list in Table 9.

**Table 9:** Elements concentration on the worn surface of steel ball.

Element abundance(wt%)	Fe	Cr	C	O	B	N	S
RSO	25.64	1.39	71.34	1.63	0	0	0
BTEB	22.38	0.59	73.69	1.68	0.53	0.88	0
BMEB	20.54	0.66	75.63	1.24	0.48	0.94	0.23

The surface morphologies were extremely rough, and the worn scar were deep and wide when only lubricated with RSO[21], and the worn morphology of lubricated with BMEB was much smoother. The relationship of wear scar depth of steel ball surface lubricated with oil samples is RSO larger than BTEB, and BTEB much larger than BMEB, the minimum wear scar area according to the smallest WSD, which corresponding to the WSD values in Fig 7. It also means that the two kinds of additives can reduce the worn of pure oil lubrication.

From Table 9, compared to pure oil, the content of ferrum and chromium elements decreased when each additive added in RSO. The ferrum, carbon and chromium elements are the basic elements of GCr15 type steel ball. Boron, nitrogen and / or sulfur elements only come from additive, and they appeared on the worn surface when lubricated with these as-prepared borate ester, it indicated that additives have been adsorbed on or occurred tribochemistry reaction with the metal surface during the friction process. Compared with RSO and BTEB, the lubricating film produced by BMEB, contributed to the better tribological performance, and it meant that sulfur element played an important antiwear efficacy during the friction process [22]. In first, additive adsorbed on the surface of friction pairs, second, the additive caused tribochemistry reactions with metal surface to form a complex boundary film which composed of inorganic sulfur-containing compounds, and it possessed good tribological performances. The high sulfur content of BMEB made it easy to form tribochemical reaction film, which could effectively prevent the direct contact of friction pairs and harder to be destroyed for its high strength under high load, and the film produced by RSO and BTEB might easy to be destroyed by pressure and mechanical force for their insufficient strength.

### XPS analysis

**Fig 10:**
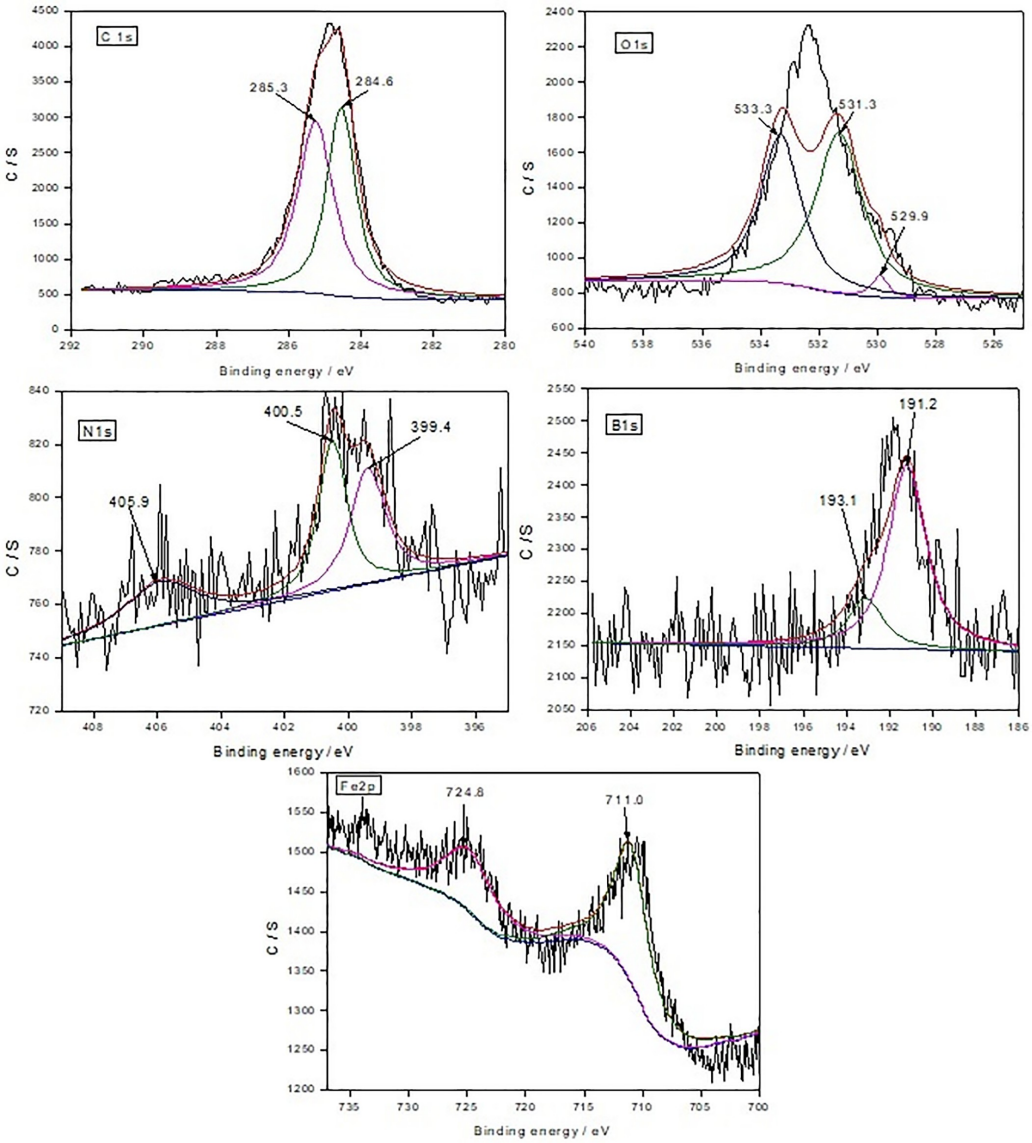
The XPS spectra of the characteristic elements of BTEB on worn surface.

**Fig 11:**
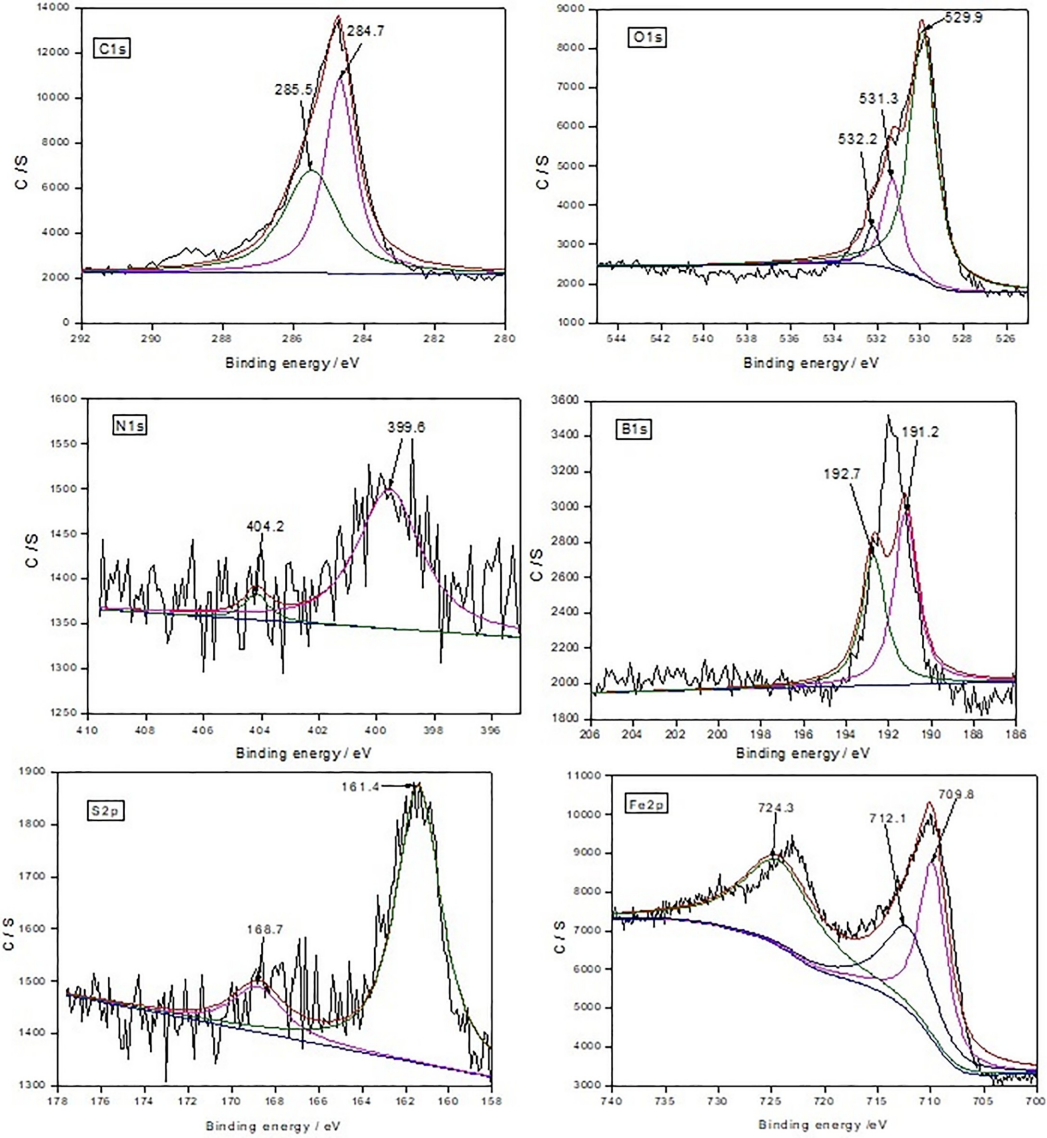
The XPS spectra of the characteristic elements of BMEB on worn surface.

The XPS spectra of characteristic element after lubricated with 0.3wt% BTEB, 0.3wt% BMEB in RSO were shown in Fig 10 and Fig 11 respectively.

In Fig 10, the C1s peaks at 284.6 and 285.3 eV correspond to the pollution of carbon, C-C/C-H and -COOH bond [23] in BTEB molecules and RSO respectively, and these indicating that BTEB or RSO has been adsorbed on the worn surface during the lubricating process, and there also exist a competitive adsorption between them.

The O1s peaks at 533.3eV, 531.3eV and 529.9eV belong to the C-O bond and iron oxide [24]. The B1s peaks at 191.2eV and 193.1eV belong to BN and B-OH or BOx bond respectively, it means that BTEB has been decomposed to form BN, BOx and B-OH group during the tribological process.

The N1s peak at 399.4eV belongs to benzotriazole ring [25], or it’s decomposed products, like azo compound [26]. The N1s peaks at 400.5 eV corresponds to organic nitrogen, and the other N1s peaks at 405.9 eV corresponds to -NO_2_ or -CN bond [27]. It indicated that BTEB occurred tribochemistry reaction, and formed a tribofilm which contained N-containing organic compound, nitro compound and /or cyanide complex.

The Fe2p peak at 711.0eV and 724.8eV belong to Fe_2_O_3_ of the Fe2p1/2 peak and Fe_3_O_4_ of the Fe2p3/2 peak respectively, and combined with the O1s peak around 530eV, the existence of FeO_x_ could be validated. For the above reason, it can be deduced that BTEB can easily form a lubricating film which included a variety of compounds containing carbon, oxygen, iron and nitrogen elements on the steel ball surface through adsorption and/or triboreaction, and it can significantly improve the tribological properties of pure oil.

In Fig 11, the C1s peaks at 284.7eV and 285.5 eV correspond to the pollution of carbon, C-C/C-H and -COOH bond [23] in BMEB molecule and RSO respectively, and there also exist a competitive adsorption between BMEB and RSO on the worn surface.

The O1s peaks at 531.3eV and 529.9eV belong to the ferrous sulfate and iron oxide [24] respectively. And the other O1s peak at 532.2eV belong to carbon oxygen bond. The B1s peaks at 191.2eV and 192.7eV belong to BN and B_2_O_3_, it also means that BMEB has occurred tribochemistical reaction to form BN and B-O containing groups.

The N1s peak at 399.6eV belongs to mercaptobenzothiazole ring[28], or it’s decomposed products, like azo compounds [26]. The other N1s peaks at 404.2 eV corresponds to -NO_2_ or -CN bond [27]. This indicated that BMEB has been adsorbed and oxized on the metal surface to form a tribofilm which contained N-containing organic compounds, nitro compounds and / or cyanide complex, and the tribofilm contributed to good friction-reducing and antiwear properties.

The Fe2p peak at 709.8eV and 724.3eV belong to Fe_2_O_3_ of the Fe2p1/2 peak and FeOOH of the Fe2p3/2 peak respectively, and combined with the O1s peak around 530eV, the existence of FeO_x_ can be validated. The other Fe2p peak at 712.1eV correspond to ferrous sulfide which agreed well with the S2p peak at 161.4eV.

The S2p peak at 161.4 eV and 168.7eV belong to the ferrous sulfide (combined with Fe2p at 712.1eV) and ferrous sulfate (combined with Fe2p at 713.6eV)[29] respectively, and this further indicated that BMEB had formed inorganic sulfur-containing film on the surface of friction pairs.

Based on the XPS analysis results, it can be concluded that BTEB and BMEB can be decompose to produce a protective film, containing BOx, FeO_x_, FeSO_4_, FeS(BMEB additive) and organic nitrogen compounds. The formed protective film can prevent the direct contact of friction pairs, therefore to increase the tribological performances of RSO[30].

The difference in chemical structure is very small between BTEB and BMEB molecules, and the only different group is benzotriazole group in BTEB, mercaptobenzoimidazoline group in BMEB, the other group all are borate ester. But they showed different tribological performances, this may be because the different functional groups played different roles in the lubricating process. Compared with BTEB molecule, the sulfur element in BMEB can form a stronger and denser protective tribofilm containing FeSO_4_, FeS by reacting with the metal surface under high load. Therefore, BMEB showed better load-carrying capacity and antiwear property in RSO than that of BTEB.

Namely, compared with these additives, base oil is first to adsorb to the metal surface because of the higher polarity. Moreover, a small amount of additive absorbed on the metal surface may influence the continuity and compactness of the oil film formed by RSO alone, resulting in destruction of the tribological properties of pure oil. In the whole lubricating process, additive molecules formed a complex boundary lubricating film on the steel ball surface, and the film improved the tribological properties of RSO.

### Antioxidation property

The IOT of different oil samples are shown in Table 10. From the Table, the IOT of RSO is 193°C, and the IOT values are 208°Cand 253°C for 0.025wt% and 0.25wt% BTEB. The IOT values are 242°C and 273°C for 0.025wt% and 0.25wt% BMEB respectively. It means that BTEB and BMEB have a certain antioxidant activity, the oxidation-resistance enhanced along with the increase of additive content, and the antioxidant activity of BTEB is stronger than that of BMEB at the same amount. Maybe the phonel- and amine-type antioxidant has better antioxidation synergy effect with benzotriazole-containing heterocyclic borate ester than with mecaptobenzoimidazole-containing borate ester in RSO.

**Table 10:** PDSC results of base oil and additive containing base oil.

Samples	IOT */*°C	*ΔT*
RSO	193	
0.025% BTEB / RSO	242	49
0.25% BTEB / RSO	273	80
0.025% BMEB / RSO	208	15
0.25% BMEB / RSO	253	60

The OIT values of PDSC were used to study oxidation kinetics of oil samples. The oxidation process of RSO is an exothermic reaction, and it will appear apparent exothermic peak [31] in the PDSC curves under heating conditions with oxygen when the RSO begins to be oxidized,. The lower OIT (t_on_) of oil samples, the worse oxidation stability. The OIT results of oil samples at different temperatures are shown in Table 11.

**Table 11:** The OIT (t_on_) of oil samples under different temperature.

*Samples*	OIT (t_on_) /min							
	*110*°C	*120*°C	*130*°C	*170*°C	*190*°C	*210*°C	*220*°C	*230*°C
RSO 0.25wt% BTEB 0.025wt% BMEB	64.31	34.50	19.30					
					>120	19.01	9.26	4.87
						9.65	5.64	3.30
0.25wt% BMEB					58.76	16.89	9.17	5.38

The OIT of BMEB is lower than that of BTEB at 0.25wt% content, indicating antioxidant capacity of BMEB (sulfur-containing) is less than that of BTEB (only contain pure nitrogen) in high temperature. From Table 11, it can be seen that the t_on_ of oil samples significantly reduced with the gradual increase of the constant temperature, which correspond to the higher temperature, the easier oxidation reaction.

The oxidation reaction of RSO and antioxidant are a free radical reaction [31]. The activation energy required for the oxidation reaction can be calculated by using the Arrhenius formula according to PDSC test results, and it can briefly investigate its oxidation mechanism. Because the chemical activation energy is a measure of the difficulty of chemistry reaction, in general, the smaller activation energy, the more easily proceeding the reaction, so it can response to the reaction degree of difficulty.

According to the Arrhenius equation:

$$\ln k=-\frac{Ea}{RT}+C$$ (1)

where k is the oxidation rate constant, and it is inversely proportional to the reaction time in the initial stages of oxidation, then

$$\ln\frac{1}{t}=-\frac{Ea}{RT}+C$$ (2)

On substituting the oxidation induction time t_on_ in Eq (2), we obtain

$$\ln t_{on}=\frac{Ea}{R}\times \frac{1}{T}+C$$ (3)

**Fig 12:**
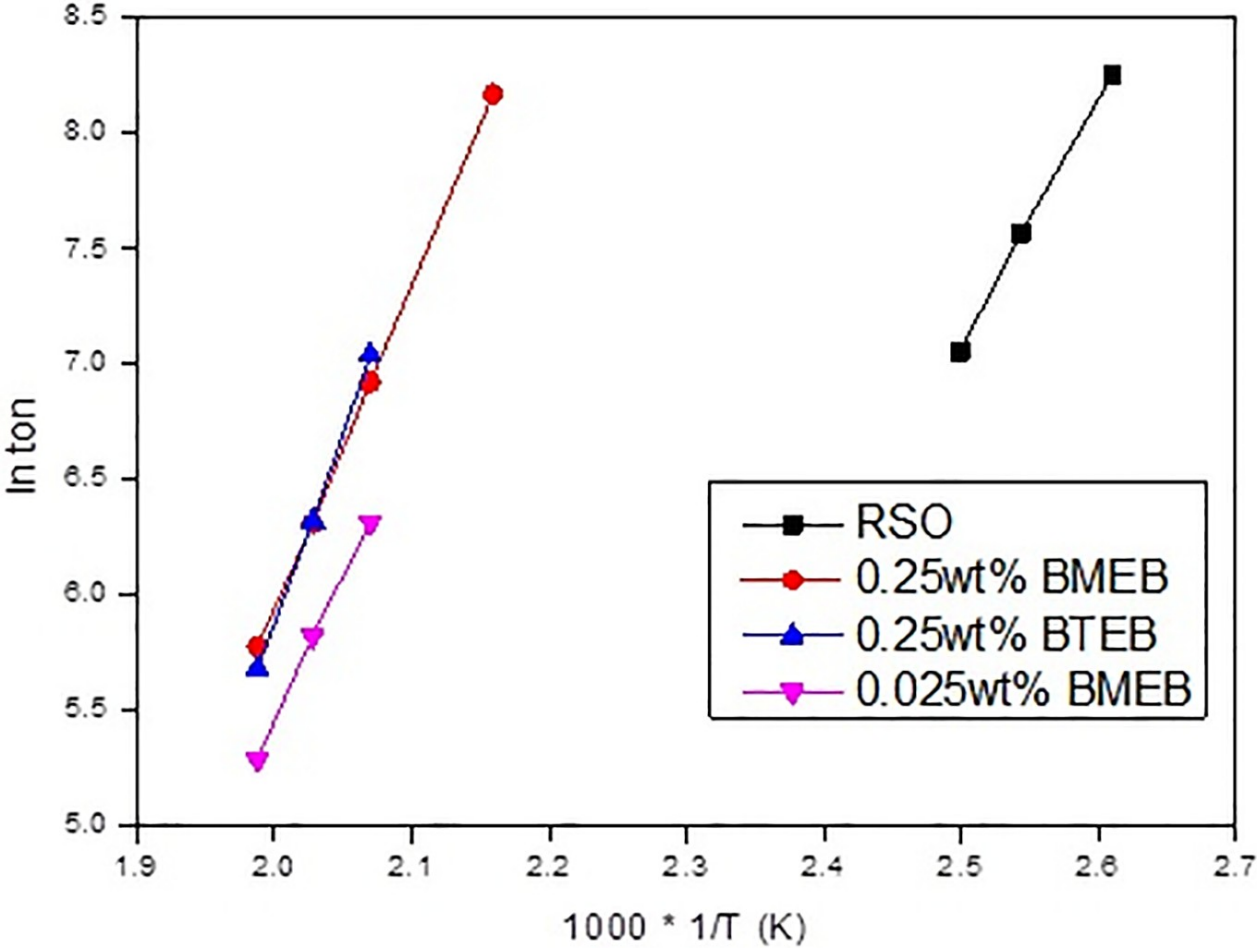
ln t_on_ ~ 1 / T curve at different temperatures.

Therefore, with ln*t_on_* for y-axis, the $\frac{1}{T}$ for x-axis, and the slope equal to $\frac{Ea}{R}$, it will obtain a straight line in theory, then to calculate the activation energy E_a_ of oxidation reaction. The results were shown in Fig 12.

In Fig 12, the resulting curves were essentially straight lines, which means that the oxidation curves of all oil samples are in accordance with the Arrhenius formula [31], and the calculated activation energy is feasible. Table 12 was shown the calculated values of oxidation activation energy of oil samples.

**Table 12:** The oxidation reaction activation energy of oil sample.

*Samples*	*RSO*	*0*.*25wt% BTEB*	*0*.*025wt% BMEB*	*0*.*25wt% BMEB*
Ea(KJ/mol)	76.951	137.605	108.321	116.311

From Table 12, it can be seen that the increase of the additive concentration, the activation energy increased, corresponding OIT value in Table 12. The BMEB and BTEB at 0.25wt% content increased the Ea of RSO by 51.15% and 78.82% respectively, and thus improve the antioxidant effect of RSO. The higher Ea of oil sample, the longer OIT, and the better antioxidation performance.

## Conclusions

The following conclusions can be drawn from the above results:

1. The synthesized BTEB and BMEB had good store stability. The hydrolysis time of BTEB, BMEB are more than 1,300 times, 1600 times than that of tributhyl borate respectively, and they are more than that of the mixed liquid of TBB and DDP. The lone electron of N atoms in heterocyclic ring was formed a coordination effect with the B atom to compensate for the shortage of electrons in the B atom and improved the hydrolysis stability of borate ester. Because the space structure of the formed of six-or seven-member ring produced by boron and nitrogen atoms is stable, and it increased steric hindrance to increase hydrolysis stability.
2. The P_*B*_ value of oil samples showed an upward trend along with the additives content increasing. When the concentration of additives were in 0.5wt%, the BTEB and BMEB enhanced the P_*B*_ value of RSO more than 60.6% and 67.7% respectively.
3. The WSD was decreased along with the increase of the BTEB and BMEB additive concentration from 0.10wt% to 1.0wt% in all applied loads, and the WSD increases with the increase of applied load. When additive concentration was at 0.5 wt %, the WSD of BTEB and BMEB reduced the WSD of RSO about 15% and 38% respectively. In high load (392N), the antiwear effect of BMEB was more superior at high concentration. The friction-reduction effect of BTEB is better than that of BMEB under 196N, the friction coefficient of BMEB is less than BTEB under 392N. In the 294N load, the friction-reducing performance of BTEB is better than that of BMEB when the adding concentration is less than 0.5wt%, while the adding concentration is greater than 0.5wt%, the friction-reducing performance of BMEB is better than BTEB, but the friction coefficient is not very different.
4. BTEB and BMEB can form a protective film on the metal surfaces which containing boron oxide, iron oxide, ferrous sulfate, ferrous sulfide (BMEB additive) and organic nitrogen compounds. BMEB shows better tribological capacities (load-carrying and antiwear) than BTEB in RSO, due to the sulfur element in BMEB, the different reaction films formed on the metal surface. The BTEB and BMEB happen tribochemical reaction with test ball surface in the friction process, generating a mixed boundary lubricating film, which is good to enhance the tribological effect of oil sample.
5. The BMEB and BTEB have some antioxidant properties, and the antioxidation effect improved with the increase of borate esters content. The antioxidant effect of BTEB is better than BMEB, this is mainly because it can improve the oxidation Ea of RSO more effectively, and the Ea of RSO was increased by 51.15% and 78.82% respectively at 0.25wt% BMEB and BTEB.
